# Motor and sensory impairment in survivors of childhood central nervous system (CNS) tumors in the St. Jude Lifetime Cohort (SJLIFE)

**DOI:** 10.1002/cam4.7422

**Published:** 2024-07-26

**Authors:** Rozalyn L. Rodwin, Fang Wang, Lu Lu, Zhenghong Li, Deo Kumar Srivastava, Nicholas S. Phillips, Raja B. Khan, Tara M. Brinkman, Kevin R. Krull, Frederick A. Boop, Gregory T. Armstrong, Thomas E. Merchant, Amar Gajjar, Leslie L. Robison, Melissa M. Hudson, Nina S. Kadan‐Lottick, Kirsten K. Ness

**Affiliations:** ^1^ Department of Pediatrics Yale University School of Medicine New Haven Connecticut USA; ^2^ Yale Cancer Center New Haven Connecticut USA; ^3^ Department of Epidemiology and Cancer Control St. Jude Children's Research Hospital Memphis Tennessee USA; ^4^ Department of Biostatistics St. Jude Children's Research Hospital Memphis Tennessee USA; ^5^ Department of Pediatrics St. Jude Children's Research Hospital Memphis Tennessee USA; ^6^ Department of Psychology and Behavioral Sciences St. Jude Children's Research Hospital Memphis Tennessee USA; ^7^ Department of Surgery St. Jude Children's Research Hospital Memphis Tennessee USA; ^8^ Department of Radiological Sciences St. Jude Children's Research Hospital Memphis Tennessee USA; ^9^ Department of Oncology St Jude Children's Research Hospital Memphis Tennessee USA; ^10^ Cancer Prevention and Control Program Georgetown Lombardi Comprehensive Cancer Center Washington DC USA

**Keywords:** central nervous system tumor, childhood cancer, cranial radiation, etoposide, motor impairment, peripheral neuropathy, platinum, quality of life, sensory impairment, survivorship, vinca alkaloid

## Abstract

**Background:**

Survivors of childhood central nervous system (CNS) tumors can develop motor and sensory impairment from their cancer and treatment history. We estimated the prevalence of motor and sensory impairment in survivors compared with controls through clinical assessment and identified associated treatment exposures and functional, quality of life (QOL), and social outcomes.

**Methods:**

Survivors of childhood CNS tumors from the St. Jude Lifetime Cohort (*n* = 378, median [range] age 24.0 [18.0–53.0] years, 43.4% female) ≥5 years from diagnosis and controls (*n* = 445, median [range] age 34.0 [18.0–70.0] years, 55.7% female) completed in‐person evaluation for motor and sensory impairment using the modified Total Neuropathy Score. Impairment was graded by modified Common Terminology Criteria for Adverse Events. Multivariable models estimated associations between grade ≥2 motor/sensory impairment, individual/treatment characteristics, and secondary outcomes (function by Physical Performance Test, fitness by physiologic cost index, QOL by Medical Outcomes Survey Short Form‐36 physical/mental summary scores, social attainment).

**Results:**

Grade ≥2 motor or sensory impairment was more prevalent in survivors (24.1%, 95% Confidence Interval [CI] 19.8%–29.4%) than controls (2.9%, CI 1.4–4.5%). Among survivors, in multivariable models, motor impairment was associated with vinca exposure <15 mg/m^2^ versus none (OR 4.38, CI 1.06–18.08) and etoposide exposure >2036 mg/m^2^ versus none (OR 12.61, CI 2.19–72.72). Sensory impairment was associated with older age at diagnosis (OR 1.09, CI 1.01–1.16) and craniospinal irradiation versus none (OR 4.39, CI 1.68–11.50). There were lower odds of motor/sensory impairment in survivors treated in the year 2000 or later versus before 1990 (Motor: OR 0.29, CI 0.10–0.84, Sensory: OR 0.35, CI 0.13–0.96). Motor impairment was associated with impaired physical QOL (OR 2.64, CI 1.22–5.72).

**Conclusions:**

In survivors of childhood CNS tumors, motor and sensory impairment is prevalent by clinical assessment, especially after exposure to etoposide, vinca, or craniospinal radiation. Treating motor impairment may improve survivors' QOL.

## 1 INTRODUCTION

Childhood cancer survivors frequently experience motor and sensory impairment, especially those treated with vinca alkaloid or platinum agents.^1^, ^2^, ^3^, ^4^, ^5^ Motor and sensory impairment can impact survivors' overall health and quality of life (QOL),^2^, ^6^, ^7^ and is amenable to rehabilitation to improve symptoms.^6^

Survivors of childhood CNS tumors are particularly at risk for motor and sensory impairment due to their multiple neurotoxic treatment exposures.^6^, ^8^, ^9^ These exposures include vinca alkaloid and platinum chemotherapy agents that are commonly associated with peripheral neuropathy,^6^ as well as CNS insults from their primary cancer and other neurotoxic treatment modalities.^8^, ^9^ However, it can be challenging to measure motor and sensory signs and symptoms that can be indicative of neuropathy in this population, and prior studies have largely relied on self‐report.^10^, ^11^ Better measurement of the prevalence and predictors of motor and sensory impairment in survivors of CNS tumors is needed to identify at‐risk survivors who would benefit from interventions and to inform upfront treatment plans for newly diagnosed patients.^12^, ^13^

The St. Jude Lifetime Cohort Study (SJLIFE) is an informative resource from which to estimate the prevalence of motor and sensory impairment in survivors of childhood CNS tumors and to evaluate individual and treatment factors associated with this outcome. The large sample of survivors with standardized in‐person assessments allows for more accurate identification of motor and sensory impairment than self‐report data in this complex population.^14^, ^15^ Among survivors of CNS tumors participating in SJLIFE, we aimed to (1) estimate the prevalence of motor and sensory impairment (motor and sensory signs and symptoms that can be indicative of neuropathy) in survivors compared to non‐first degree relative controls without a history of pediatric cancer, (2) identify individual and treatment‐related factors associated with risk of motor and sensory impairment, and (3) measure associations between motor and sensory impairment and physical function, QOL, and social attainment.

## 2 MATERIALS AND METHODS

### 2.1 Participants

**FIGURE 1:**
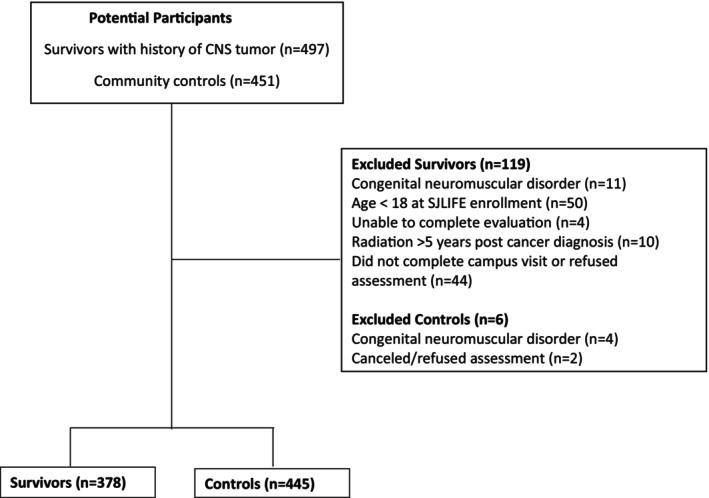
Consort diagram.

Participants were survivors of CNS tumors from SJLIFE, a retrospective cohort of childhood cancer survivors diagnosed and treated at St. Jude Children's Research Hospital between 1962 and 2012 with prospective comprehensive on‐site medical evaluations. The SJLIFE study design and methodology have been previously described.^14^, ^15^ Participants in the current analysis had a CNS tumor treated at St. Jude, completed a functional assessment, were ≥5 years from their cancer diagnosis and ≥18 at the time of evaluation. Individuals who did not complete a campus visit or refused assessment (*n* = 44), or had a congenital neuromuscular disorder (*n* = 11) or physical or neurocognitive impairment that prevented completion of a functional assessment (*n* = 4), were excluded from the analysis (Figure 1). Survivors who received craniospinal irradiation (CSI) more than 5 years after their cancer diagnosis were also excluded to avoid confounding from neurotoxic exposures unrelated to their initial treatment. Of 497 CNS tumor survivors, 378 met eligibility criteria. A control group consisting of non‐first‐degree relatives of St. Jude patients was frequency matched to all participants in the SJLIFE cohort by age (within 5 years), sex, and race; 445 controls met eligibility criteria and were included in the analysis. The study was approved by the St. Jude Institutional Review Board, and all participants provided written informed consent.

### 2.2 Outcomes

#### 2.2.1 Primary outcomes

Motor and sensory impairment, which included motor and sensory signs and symptoms indicative of neuropathy, was identified by exercise physiologist or physical therapist evaluation using the modified Total Neuropathy Score (mTNS).^16^ Severity was graded using the modified version of the National Cancer Institute's Common Terminology Criteria for Adverse Events (CTCAE) version 4.03 used for motor and sensory neuropathy, as previously described in SJLIFE (Table S1).^17^ For multivariable analyses, this outcome was dichotomized into grades 0–1 and grades ≥2 (moderate to severe impairment) to assess factors associated with motor and sensory impairment that limit activities of daily living.^18^

Motor impairment was defined by motor symptoms and physical examination with presence of distal weakness by manual muscle examination, using the motor examination component of the mTNS.^16^ Additional presence of hand or foot weakness >1.5 standard deviation (SD) below community average was used to define grade ≥2 motor impairment. Hand strength was measured using a hand‐held dynamometer (System 4 dynamometer; Biodex Medical Systems, Shirley, NY)^19^ administered by a physical therapist/exercise physiologist trained to encourage maximum effort, and the maximum value of two sequential trials was used for analysis.^20^, ^21^ Foot strength was calculated using peak torque of ankle dorsiflexion at 60/second (Biodex System 3.0, Biodex Medical Systems, Shirley, NY). The average value of the best attempt of each side was included for analysis when bilateral measures were available.

Sensory impairment was defined by sensory symptoms (including pain, numbness, or paresthesia) and physical examination with impaired pin or vibration sensibility, based on the sensory examination components of the mTNS.^16^ Additional presence of slowed writing speed or impaired balance in the absence of nystagmus, dysarthria, dysmetria, or dysdiadochokinesia was used to upgrade sensory impairment to grade ≥2.^22^ Slowed writing speed was evaluated by the first item in the Physical Performance Test (PPT) and was impaired if writing time for a short sentence was >10 seconds.^23^ Balance was measured using the Sensory Organization Test (SOT) (NeuroCom SMART EquiTest, Natus Medical, Pleasanton, CA). The percentage of time participants spent upright in a 12.5° sway envelope under six conditions was recorded, and a composite score ≤70 was considered impaired.^24^

#### 2.2.2 Secondary outcomes

##### Functional outcomes

Physical function was measured by the composite score on the Physical Performance Test (PPT),^25^ with a score >1.5 SD below the mean of community matched controls considered impaired.^23^ The first item, writing time, was excluded from scoring as it was included in the definition of sensory impairment. Fitness was assessed by calculating the physiologic cost index (PCI) using the formula PCI = (maximum heart rate – resting heart rate)/gait speed, and was considered impaired if >1.5 SD above the mean of community matched controls.^25^

##### QOL/Social Attainment

QOL was assessed using the validated Medical Outcomes Survey Short Form‐36 (SF‐36) version 2.^26^ Physical component summary scale and mental component summary scale scores were calculated, and *t*‐scores ≤40 were considered impaired.^25^ Social attainment was assessed in survivors ≥25 years of age at evaluation by survey responses regarding current employment status (employed [working full‐time, part‐time, student, or caring for home/family] versus unemployed [unemployed looking for work, unable to work, or retired]), educational attainment (less than college degree versus college or higher), and dependent living status (dependent [living with parent, siblings, or other non‐child relatives] versus independent [living with spouse or alone]).

### 2.3 Individual and disease characteristics

Individual characteristics obtained from surveys included age at assessment, sex, race, and ethnicity. Disease and treatment factors were abstracted from medical records using a structured protocol.^14^ Analyzed treatment exposures and disease characteristics included vinca alkaloid exposure (yes/no and by dose; 0 mg/m^2^, 0 to ≤15 mg/m^2^, and >15 mg/m^2^), platinum exposure (yes/no and by dose; 0 mg/m^2^, 0 to ≤1383 mg/m^2^, and >1383 mg/m^2^), cisplatin exposure (yes/no), etoposide exposure (yes/no and by dose; 0 mg/m^2^, 0 to ≤2036 mg/m^2^, >2036 mg/m^2^) history of radiation (none, cranial only, or CSI), CSI by dose (0 cGy, 0 to ≤3295 cGy, and >3295 cGy), maximum radiation dose to the posterior fossa (by tertile of exposure), tumor location (supratentorial, posterior fossa, or spinal cord),^27^ type of surgery (gross or near total resection, partial resection, or no surgery/biopsy only), history of hydrocephalus,^14^ age at diagnosis, and treatment era (before 1990, 1990–1999, 2000 and later). Treatment factors were also grouped by modality (no treatment or surgery only, focal radiation, chemotherapy [with or without focal radiation], CSI, CSI with chemotherapy). The cut‐point for vinca alkaloid, platinum, etoposide, and CSI dose was based on examination of receiver operating curves with the outcome grade ≥2 motor impairment.

### 2.4 Other covariates

#### 2.4.1 Comorbid conditions

Chronic conditions were classified using the modified version of CTCAE 4.03 specific to childhood cancer survivors.^17^, ^28^ For the current analysis, grade ≥2 chronic conditions included neurologic (hemiplegia/paraplegia, stroke, nerve root disorder, or cerebellar dysfunction), respiratory (asthma, chronic obstructive pulmonary disease, restrictive lung disease), cardiovascular (cardiomyopathy, heart failure, peripheral vascular disease), and endocrine (diabetes, obesity) conditions, as well as hearing and visual impairment.^21^, ^29^, ^30^ Survivor‐report of receipt of physical therapy (PT) in the past year was also included as a covariate, as this can modify CIPN symptoms.^31^

#### 2.4.2 Neurocognitive function

Neurocognitive function was included as a covariate in models examining associations between motor and sensory impairment and social attainment.^32^ General intelligence was measured using the Wechsler Abbreviated Scale of Intelligence (WASI).^33^ Additional neurocognitive domains included executive function (cognitive flexibility by Trail Making Part B, cognitive fluency by FAS word association, and working memory by digit backward)^33^ and math and reading academic achievement by Woodcock‐Johnson‐III achievement test.^34^ Motor and visual/motor processing speed assessed by grooved pegboard and digit‐symbol coding were included as covariates in social attainment and physical function models.^33^, ^35^ All scores were converted to *z*‐scores using standardized age/sex‐based norms as previously described in this cohort.^36^

### 2.5 Analyses

Individual characteristics, motor impairment, sensory impairment, and physical function, QOL, and social attainment outcomes were compared between survivors and controls using chi‐square or Wilcoxon rank test as appropriate.

Associations between individual characteristics, treatment exposures, disease characteristics, and grade ≥2 motor and sensory impairment were estimated using logistic or multinomial regression models. Separate regression models, adjusted for chronic conditions listed above, estimated associations between motor and sensory impairment and clinically assessed physical function, self‐reported QOL, and social attainment (in survivors ages 25 years and older). The LASSO method was used to determine variables included in the final models, and logistic or multinomial regression models were re‐run to estimate associations between outcomes and predictor variables.^37^, ^38^ All analyses were performed using SAS (version 9.4, Cary, North Carolina).

## 3 RESULTS

### 3.1 Description of study population

A total of 378 survivors and 445 age, sex, and race‐matched controls were included in the analysis (Figure 1). Characteristics of survivors and controls, and survivor participants and non‐participants, are provided in Table 1. The most common primary cancer diagnoses among survivor participants were astroglial tumors (49.2%) and medulloblastoma (27.0%). Among participating survivors, 38.6% received chemotherapy (26.5% vinca alkaloid, 33.1% platinum agent, 13.8% etoposide), and 68.5% of survivors received radiotherapy (36.0% cranial, 32.5% craniospinal).

**TABLE 1:** Characteristics of survivors and age‐, sex‐, race‐matched community controls.

	Survivors (*n* = 378)	Controls (*n* = 445)	*p* Value^a^	Non‐participant survivors (*n* = 44)	*p* Value^b^
Age in years (median, range)	24.0 (18.0–53.0)	34.0 (18.0–70.0)	<0.001	30.0 (19.0–52.0)	<0.0001
Sex (*n*,%)			<0.001		0.40
Female	164 (43.4)	248 (55.7)		22 (50.0)	
Male	214 (56.6)	197 (44.3)		22 (50.0)	
Race/ethnicity (*n*, %)			<0.001		0.65
White, non‐hispanic	306 (81.0)	364 (81.8)		38 (86.4)	
Hispanic	5 (1.3)	14 (3.1)		1 (2.3)	
Black	60 (15.9)	31 (7.0)		4 (9.1)	
Other	7 (1.9)	36 (8.1)		1 (2.3)	
Physical performance test (*n*, %)					
Impaired	133 (35.2)	17 (3.8)	<0.001		
Not impaired	245 (64.8)	428 (96.2)			
Physiologic cost index (*n*, %)					
Impaired	33 (8.7)	20 (4.5)	0.014		
Not impaired	345 (91.3)	425 (95.5)			
Education (*n*, %)					0.93
High school or less	154 (40.7)	82 (18.4)	<0.001	17 (38.6)	
Post high school training	146 (38.6)	120 (27.0)		14 (31.8)	
College or higher	78 (20.6)	230 (51.7)		8 (18.2)	
Missing/unknown	0 (0.0)	13 (2.9)		5 (11.4)	
Employment (*n*, %)			<0.001		0.45
Employed	228 (60.3)	371 (83.4)		26 (59.1)	
Unemployed	149 (39.4)	60 (13.5)		13 (29.5)	
Missing/unknown	1 (0.3)	14 (3.1)		5 (11.4)	
Independent living (*n*, %)^c^			<0.001		0.59
Independent	167 (44.2)	375 (84.3)		19 (43.2)	
Dependent	211 (55.8)	58 (13.0)		20 (45.4)	
Missing/unknown	0 (0.0)	12 (2.7)		5 (11.4)	
Impaired quality of life (*n*, %)					
Physical summary scale score impaired	61 (17.5)	24 (5.6)	<0.001	8 (18.2)	0.54
Mental summary scale score impaired	57 (16.4)	57 (13.3)	0.24	7 (15.9)	0.69
Reported physical therapy use in past 2 years (*n*, %)			0.008		0.04
Yes	63 (16.7)	43 (9.7)		2 (4.55)	
No	284 (75.1)	354 (79.6)		35 (79.6)	
Missing	31 (8.20)	48 (10.8)		7 (15.9)	
Reported current gabapentin use	2 (0.5)	0 (0)	0.21	2 (4.6)	0.06
**Disease characteristics**					
Age at diagnosis (years, median, range)	8.7 (4.6)				
Treatment era					
<1990	57 (15.1)				
1990–1999	206 (54.5)				
After 2000	115 (30.4)				
Time since diagnosis (years, median, range)	15.6 (8.0–42.3)			23.5 (6.2–47.1)	<0.0001
Tumor Histology					<0.0001
Astroglial tumor	186 (49.2)			20 (45.5)	
Suptratentorial astroglial tumor	122 (32.3)			12 (27.3)	
Posterior fossa astroglial tumor	62 (16.4)			7 (15.9)	
Spinal Cord	2 (0.5)			1 (2.3)	
Craniopharyngioma	29 (7.7)			1 (2.3)	
Ependymoma	41 (10.9)			2 (4.6)	
Germ cell tumor	12 (3.2)			6 (13.6)	
Medulloblastoma	102 (27.0)			12 (27.3)	
Other	8 (2.1)			3 (6.8)	
Tumor Location					0.75
Posterior fossa	185 (48.9)			19 (43.2)	
Supratentorial	187 (49.5)			24 (54.5)	
Spinal cord	6 (1.6)			1 (2.3)	
Surgery					0.96
Gross or near total	215 (56.9)			26 (59.1)	
Partial/other	92 (24.3)			10 (22.7)	
No surgery/biopsy	71 (18.8)			8 (18.2)	
Hydrocephalus	124 (32.8%)			13 (29.5)	0.66
Received chemotherapy	146 (38.6)			16 (36.4)	0.77
Vinca alkaloid					
Any	100 (26.5)			9 (20.5)	0.39
Dose mg/m^2^ (median, range)	10.9 (1.2–125.0)			7.5 (3.4–42.5)	0.52
Platinum^d^					
Any	125 (33.1)			14 (31.8)	0.87
Dose mg/m^2^ (median, range)	304.6 (73.0–11647.0)			714.8 (176.9–8827.3)	0.18
Etoposide					
Any	52 (13.8)			10 (22.7)	0.11
Dose mg/m^2^ (median, range)	983.9 (237.8, 3623.5)			940.7 (692.9–2620)	0.57
Radiation type (*n*,%)					0.24
Cranial only	136 (36.0)			10 (22.7)	
Craniospinal	123 (32.5)			20 (45.5)	
No radiotherapy	119 (31.5)			14 (31.8)	
Radiation dose to posterior fossa, Gy, (median, range)	55.0 (0.2–106)			54.0 (46.0–59.0)	0.16
Treatment modality (*n*,%)^e^					0.95
No treatment	106 (28.0)			14 (31.8)	
Focal radiation only	110 (29.1)			11 (25.0)	
Chemotherapy with or without focal radiation	67 (17.7)			7 (15.9)	
Craniospinal radiation only	19 (5.0)			3 (6.8)	
Craniospinal irradiation and chemotherapy	76 (20.1)			9 (20.5)	

^a^ *p* Value is describing comparison between survivors and controls.

^b^ *p* Value is describing comparison between survivor participants and non‐participants.

^c^ Independent living = lives with spouse, alone, or roommate, Dependent living = Lives with parent, siblings, or other non‐child relative.

^d^ Cumulative dose of cisplatin and carboplatin.

^e^ Each treatment modality included participants who also received surgery.

### 3.2 Prevalence of motor and sensory impairment

Among survivors, 91 (24.1%; 95% CI 19.8%–29.4%) had any grade ≥2 motor or sensory impairment (motor only 27 [7.1%], sensory only 40 [10.6%], both 24 [6.4%]) (Table 2). Survivors were more likely than controls to have grade ≥2 motor impairment (13.5% survivors [grade 2 = 7.7%, grade 3 = 5.8%] versus 0.9% controls, *p* < 0.001) or sensory impairment (16.9% survivors [grade 2 = 10.9%, grade 3 = 6.1%] versus 2.3% controls, *p* < 0.001, Table S2). When examining prevalence of motor and sensory impairment by tumor histology, ependymoma survivors had the highest prevalence of motor impairment (22.0%), and medulloblastoma survivors had the highest prevalence of sensory impairment (33.3%, Table S3).

**TABLE 2:** Prevalence of motor and sensory impairment in survivors and community matched controls.

	Survivors *n* (%)	95% CI	Controls *n* (%)	95% CI	*p* Value^a^
Motor impairment					<0.001
Grade 0	307 (81.2)	77.3–85.2	440 (98.9)	97.9–99.9	
Grade 1	19 (5.0)	2.8–7.2	1 (0.2)	0.0–0.7	
Grade ≥2	51 (13.5)	10.1–16.9	4 (0.9)	0.0–1.8	
Missing	1 (0.3)	0.0–0.8	0 (0.0)		
Sensory impairment					
Grade 0	273 (72.2)	67.7–76.7	387 (87.4)	83.8–90.1	<0.001
Grade 1	39 (10.3)	7.3–13.4	46 (10.4)	7.5–13.2	
Grade ≥2	64 (16.9)	13.2–20.7	10 (2.3)	0.9–3.6	
Missing	2 (0.5)	70.0–1.3	2 (0.5)	0.0–1.1	
Overall motor or sensory impairment					<0.001
Grade 0–1	285 (75.4)	71.1–79.7	430 (96.6)	95.0–98.3	
Grade ≥2	91 (24.1)	19.8–29.4	13 (2.9)	1.4–4.5	
Grade ≥2 motor only	27 (7.1)	4.6–9.7	3 (0.7)	0–1.4	
Grade ≥2 sensory only	40 (10.6)	7.5–13.7	9 (2.0)	0.7–3.3	
Grade ≥2 both	24 (6.4)	3.9–8.8	1 (0.2)	0–0.7	
Missing	2 (0.5)	0–1.3	2 (0.5)	0–1.1	

^a^ *p* Values were calculated using Fisher's Exact test with missing values excluded. *p* values remained <0.001 when comparing grade 0–1 versus grade ≥2 impairment.

### 3.3 Individual and treatment characteristics associated with motor and sensory impairment

Among survivors, treatment factors associated with grade ≥2 motor impairment in multivariable models included vinca alkaloid exposure ≤15 mg/m^2^ versus none (OR = 4.38, 95% CI 1.06–18.08), and etoposide exposure >2036 mg/m^2^ versus none (OR = 12.61 95% CI 2.19–72.72, Table 3). Individual and treatment factors associated with grade ≥2 sensory impairment in multivariable models included age at diagnosis (OR = 1.09, 95% CI 1.01–1.16) and CSI versus no radiation (OR = 4.39, 95% CI 1.68–11.50). CSI dose was not associated with sensory impairment. Later treatment era, in 2000 or later (versus before 1990), was associated with a lower odds of motor (OR = 0.29, 95% CI 0.10–0.84) and sensory (OR = 0.35, 95% CI 0.13–0.96) impairment. Platinum exposure and cisplatin exposure alone were not associated with motor or sensory impairment. When assessing associations between treatment modality and impairment (No treatment, focal radiation, chemotherapy, CSI, or CSI and chemotherapy), treatment modality was not associated with grade ≥2 motor impairment, but the combination of CSI and chemotherapy was associated with grade ≥2 sensory impairment (OR 5.72, 95% CI 2.49–13.14) when compared with no treatment or surgery only (Table S4).

**TABLE 3:** Treatment and disease characteristics associated with grade ≥2 motor and sensory impairment in multivariable models.^a^

	Motor impairment (*N* = 51)			Sensory impairment (*N* = 64)		
	*n* (%)	OR (95% CI)	*p* Value	*n* (%)	OR (95% CI)	*p* Value
Age at diagnosis^b^ (years)		1.01 (0.94–1.08)	0.741		**1.09 (1.01–1.16)**	**0.019**
Treatment era^b^						
Before 1990	14 (27.5)	1.00		15 (23.4)	1.00	
1990–1999	28 (54.9)	0.50 (0.22–1.15)	0.105	30 (46.9)	0.47 (0.20–1.07)	0.072
2000 and later	9 (17.7)	**0.29 (0.10–0.84)**	**0.023**	19 (29.7)	**0.35 (0.13–0.96)**	**0.042**
Radiation		^c^	^c^			
None	13 (25.5)			9 (14.1)	1.00	
Cranial	14 (27.5)			14 (21.9)	1.30 (0.53–3.21)	0.565
Craniospinal	24 (47.1)			41 (64.1)	**4.39 (1.68‐11.50)**	**0.003**
Vinca alkaloid^d^		^d^	^d^			
No	34 (66.7)			33 (51.6)	1.00	
Yes	17 (33.3)			31 (48.4)	2.01 (0.73‐5.50)	0.175
Vinca alkaloid^e^					^d^	^d^
None	34 (66.7)	1.00		33 (51.6)		
≤15 mg/m^2^	10 (19.6)	**4.38 (1.06‐18.08)**	**0.041**	23 (35.9)		
>15 mg/m^2^	7 (13.7)	1.80 (0.54‐5.99)	0.340	8 (12.5)		
Platinum^b^						
None	31 (60.8)	1.00		31 (48.4)	1.00	
≤1383 mg/m^2^	13 (25.5)	0.24 (0.05‐1.21)	0.083	28 (43.8)	1.01 (0.28–3.68)	0.988
>1383mg/m^2^	7 (13.7)	0.50 (0.11‐2.19)	0.355	5 (7.8)	1.04 (0.25–4.35)	0.957
Etoposide^b^						
None	37 (72.6)	1.00		52 (81.3)	1.00	
≤2036 mg/m^2^	9 (17.7)	3.02 (0.85‐10.72)	0087	9 (14.1)	0.57 (0.19‐1.68)	0.306
>2036 mg/m^2^	5 (9.8)	**12.61 (2.19‐72.72)**	**0.005**	3 (4.7)	1.24 (0.22‐6.89)	0.810

*Note*: Bold value indicates *p*‐value <0.05.

^a^ LASSO model selection was used to determine variables included in the final model.

^b^ Age at diagnosis, treatment era, platinum dose, and etoposide dose were not selected for motor or sensory neuropathy models, but were forced in to estimate associations.

^c^ Blank cell indicates variable not selected for this model.

^d^ Vinca alkaloid was included as dichotomous variable in sensory model and categorized by cumulative exposure dose in motor model based on examination of distribution and examination of receiver operating characteristic curves.

^e^ Vinca alkaloid dose was not selected for motor impairment model, but was forced in to estimate associations.

### 3.4 Function, QOL, and social attainment outcomes associated with motor and sensory impairment

In multivariable models, neither motor nor sensory impairment were associated with impaired physical function or fitness; however, lower visual/motor processing (OR 0.22, 95% CI 0.14–0.37) and motor processing (OR 0.70, 95% CI 0.54–0.91) *z*‐scores were associated with impaired physical function (Table 4). Grade ≥2 motor impairment was associated with impaired physical QOL (OR 2.64, 95% CI 1.22–5.72) but was not associated with mental QOL or social attainment. Grade ≥2 sensory impairment was not associated with QOL or social attainment (Table 5).

**TABLE 4:** Association of grade ≥2 motor and sensory impairment with physical function outcomes in survivors in multivariable models.^a^

Motor impairment				
	Impaired physical function^b^		Impaired fitness^c^	
	OR (95% CI)	*p* Value	OR (95% CI)	*p* Value
Motor impairment (yes/no)	1.77 (0.73–4.31)	0.206	^d^	^d^
Visual/motor processing^e^ (*z*‐score)	**0.22 (0.14–0.37)**	**<0.001**	^d^	^d^
Motor processing^e^ (*z*‐score)	**0.70 (0.54–0.91)**	**0.007**	^d^	^d^

Sensory Impairment				
	Impaired physical function^b^		Impaired fitness^b^	
	OR (95% CI)	*p* Value	OR (95% CI)	*p* Value
Sensory impairment (yes/no)	1.76 (0.86–3.59)	0.122	^d^	^d^
Visual/motor processing^e^ (*z*‐score)	**0.22 (0.13–0.36)**	**<0.001**	^d^	^d^
Motor processing^e^ (*z*‐score)	**0.72 (0.55–0.93)**	**0.011**	^d^	^d^

*Note*: Bold value indicates *p*‐value <0.05.

Abbreviation: IQ; intelligence quotient.

^a^ Model selection was performed using the LASSO method to determine variables included in the final model. For impaired physical function motor and sensory impairment were not selected for the model but were included to estimate associations.

^b^ Impaired physical function measured by Physical Performance Test with impairment defined as a score >1.5 standard deviations below the community average.

^c^ Impaired fitness measured by Physiologic Cost Index with impairment defined as a score >1.5 standard deviations above the community average.

^d^ Blank cell indicates variable not selected for given outcome in adjusted model.

^e^ Motor processing refers to performance on grooved pegboard test, and visual motor processing refers to performance on digit‐symbol coding test.

**TABLE 5:** Association of grade ≥2 motor and sensory impairment and sensory impairment with QOL and role attainment outcomes in survivors in multivariable models.^a^

Motor impairment										
	Impaired mental QOL^b^		Impaired physical QOL^b^		Employed^c^		Independent living^c^, ^d^		College or higher^c^	
	OR (95% CI)	*p* Value	OR (95% CI)	*p* Value	OR (95% CI)	*p* Value	OR (95% CI)	*p* Value	OR (95% CI)	*p* Value
Motor impairment (yes/no)	^e^	^e^	**2.64 (1.22–5.72)**	**0.014**	0.38 (0.13–1.10)	0.074	0.96 (0.35–2.64)	0.930	1.46 (0.36–5.93)	0.598
Verbal fluency (*z*‐score)	^e^	^e^	0.81 (0.58–1.13)	0.220	^e^	^e^	^e^	^e^	^e^	^e^
Full scale IQ (*z*‐score)	^e^	^e^	0.76 (0.50–1.16)	0.200	**1.96 (1.23–3.14)**	**0.005**	**1.68 (1.12–2.53)**	**0.013**	1.43 (0.79–2.59)	0.238
Math academic performance (*z*‐score)	^e^	^e^	0.86 (0.60–1.25)	0.430	^e^	^e^	^e^	^e^	**3.58 (2.06–6.19)**	**<0.001**
Visual/motor processing^f^ (*z*‐score)	^e^	^e^	^e^	^e^	^e^	^e^	**1.60 (1.07–2.39)**	**0.023**	^e^	^e^
Motor processing^f^ (*z*‐score)	^e^	^e^	^e^	^e^	^e^	^e^	^e^	^e^	1.07 (0.78–1.46)	0.671
Cognitive flexibility (*z*‐score)	^e^	^e^	^e^	^e^	**1.39 (1.08–1.79)**	**0.010**	^e^	^e^	^e^	^e^
Verbal fluencey (*z*‐score)	^e^	^e^	^e^	^e^	^e^	^e^	^e^	^e^	1.34 (0.87–2.06)	0.187

Sensory impairment										
	Impaired mental QOL^b^		Impaired physical QOL^b^		Employed		Independent living^b^		College or higher	
	OR (95% CI)	*p* Value	OR (95% CI)	*p* Value	OR (95% CI)	*p* Value	OR (95% CI)	*p* Value	OR (95% CI)	*p* Value
Sensory Impairment (yes/no)	^e^	^e^	1.46 (0.70–3.04)	0.318	0.55 (0.29–1.03)	0.062	0.88 (0.38–2.09)	0.779	0.55 (0.19–1.56)	0.259
Verbal fluency (*z*‐score)	^e^	^e^	0.76 (0.55–1.07)	0.112	^e^	^e^	1.14 (0.79–1.64)	0.478	1.26 (0.82–1.94)	0.284
Full scale IQ (*z*‐score)	^e^	^e^	0.76 (0.50–1.15)	0.195	**1.92 (1.21–3.06)**	**0.006**	1.23 (0.75–2.01)	0.418	1.50 (0.84–2.69)	0.174
Math academic performance (*z*‐score)	^e^	^e^	0.85 (0.59–1.23)	0.383	^e^	^e^	1.48 (0.98–2.24)	0.063	**3.58 (2.07–6.19)**	**<0.001**
Visual/motor processing^f^ (*z*‐score)	^e^	^e^	^e^	^e^	^e^	^e^	1.42 (0.93–2.18)	0.103	^d^	^d^
Motor Processing^f^ (*z*‐score)	^e^	^e^	^e^	^e^	^e^	^e^	^d^	^d^	1.01 (0.75–1.37)	0.933
Cognitive flexibility (*z*‐score)	^e^	^e^	^e^	^e^	**1.42 (1.11–1.82)**	**0.006**	^d^	^d^	^d^	^d^

*Note*: Bold value indicates *p*‐value <0.05.

Abbreviation: QOL, quality of life.

^a^ LASSO model selection was used to determine variables included in the final model. Motor impairment was not selected for employment, independent living, or education outcomes and was included to estimate associations. Sensory impairment was not selected for any outcomes but was included to estimate associations.

^b^ Impaired mental and physical QOL defined as t‐score ≤ 40 on mental and physical component summary scales of the Medical Outcomes Survey Short Form‐36.

^c^ Only survivors ages ≥25 years at assessment (*n* = 183) were included in model selection and final models for social attainment outcomes.

^d^ Independent living = lives with spouse, alone, or roommate, Dependent living = Lives with parent, siblings, or other non‐child relative.

^e^ Blank cell indicates variable not selected for given outcome in adjusted model.

^f^ Motor processing refers to performance on grooved pegboard test, and visual motor processing refers to performance on digit‐symbol coding test.

## 4 DISCUSSION

Our cross‐sectional study of 378 long‐term survivors of childhood CNS tumors and 445 community controls quantifies the prevalence of motor and sensory impairments, using a standardized clinical assessment, in long‐term survivors of childhood CNS tumors and examines their association with treatment exposures and other functional, QOL, and social attainment outcomes. In survivors of childhood CNS tumors, we demonstrated that motor or sensory impairment is prevalent in 24% of survivors by in‐person evaluation and identified a novel association of etoposide and motor impairment. We also found that motor impairment is associated with impaired QOL.

This study was the first to demonstrate an association between etoposide exposure and motor impairment in long‐term survivors of CNS tumors, with a 12‐fold increased odds of motor impairment in survivors treated with ≥2036 mg/m^2^ of etoposide compared to those who were not exposed. Etoposide causes cell death by blocking an enzyme involved in DNA synthesis.^39^ In animal models, etoposide has led to axonal changes in large myelinated fibers consistent with neuropathy,^40^ and has been associated with hind limb paralysis.^41^ Etoposide has also been associated with neuropathy in children receiving treatment with vincristine.^42^ Among 67 children treated for non‐CNS cancers, etoposide exposure and vinca alkaloid exposure, compared to vinca alkaloid alone, were associated with worse neuropathy, measured by the pediatric‐modified Total Neuropathy Score at 6 months post‐therapy.^42^ We expanded on these findings by demonstrating that etoposide is associated with motor impairment in a dose‐dependent fashion in survivors a median of 15 years post‐diagnosis. In post‐hoc analyses, we also examined whether interactions between etoposide and vinca alkaloid and etoposide and radiation exposure contributed to these findings but found no significant interactions. This finding can have important implications for clinical care since there are currently no recommendations to screen for motor or sensory impairment following etoposide exposure.^43^ Monitoring motor function for survivors of CNS tumors following etoposide exposure should be considered as part of survivorship care so that they can receive rehabilitation services, and this association should be further explored in populations of non‐CNS tumor survivors.

Another key finding of this study is the high prevalence of motor or sensory impairment in 24.1% of CNS tumor survivors and the association of motor impairment with vinca alkaloid but not platinum exposure. We found survivors exposed to vinca alkaloids at a dose up to 15 mg/m^2^ were more likely to have motor impairment than survivors who were not exposed to vinca alkaloids.^44^, ^45^ Vinca alkaloids can cause both motor and sensory impairment through inhibition of microtubule function in peripheral nerve axons,^2^, ^6^, ^46^ and have been associated with motor impairment in long‐term survivors of non‐CNS tumors.^2^, ^47^ Our findings differ from prior studies that did not find associations of chemotherapy with motor deficits in CNS tumor survivors.^44^, ^45^ In at least 5‐year survivors of childhood CNS tumors from the Childhood Cancer Survivor Study, there was no association between vinca alkaloid or platinum chemotherapy and self‐reported motor impairment (including weakness or difficulty moving arms or legs), but these studies were limited by self‐report.^10^, ^44^ A study that evaluated physical limitations in 78 survivors of childhood CNS tumors treated from 1970 to 2000 demonstrated 20.5% of survivors had loss of protective sensation, and 20.5% had hand weakness that were not associated with treatment with vinca or platinum chemotherapy.^45^ It is possible our findings differ from the prior studies due to the use of clinical assessments in a larger sample. We did not find an association between the higher dose of vinca alkaloid and motor impairment, but it is possible that this was due to the limited sample size in this group. Regardless, the findings highlight the need for interventions, such as rehabilitation services,^48^ to minimize motor and sensory deficits in survivors of CNS tumors since nearly a quarter will experience motor or sensory impairment.

In addition to the association of vinca and etoposide exposure with motor impairment, we also found older age at diagnosis and exposure to CSI were associated with an increased risk of sensory impairment, while later treatment decade was associated with a lower risk of motor and sensory impairment in long‐term survivors of CNS tumors. Radiation can cause direct nerve damage or nerve root compression from fibrosis of surrounding structures that can occur years after radiation and may explain the association of spinal radiation with sensory impairment in this study.^49^ Older age at time of treatment has been previously described as a risk factor for developing neuropathy during treatment in non‐CNS tumor patients and may have contributed to the association of age with sensory impairment in our study.^42^, ^50^ These findings suggest survivors of CNS tumors who are treated at an older age and treated with CSI may especially benefit from close monitoring for sensory deficits during survivorship care. The association of a later treatment decade with a lower risk of motor or sensory impairment likely reflects less toxic treatment protocols in later decades.^51^ A limitation of this cohort is that there were fewer survivors treated after the year 2000, which may limit the generalizability of our findings to recent survivors. However, our findings still inform care for survivors of CNS tumors currently in survivorship follow‐up, and future studies should focus on confirming these findings in more recent survivors.

We also found that motor and sensory deficits were clinically meaningful outcomes, highlighting the need for interventions to improve motor and sensory deficits. Grade ≥2 motor impairment was associated with patient‐report of impaired physical QOL, with more than a 2‐fold increased risk of impaired physical QOL among survivors with motor impairment compared with survivors without motor impairment, even when adjusting for other chronic conditions. Minimizing motor deficits may therefore be a target for intervention to improve QOL in survivors of CNS tumors. Physical therapy intervention has been reported to improve motor symptoms in children with acute lymphoblastic leukemia.^31^, ^52^, ^53^, ^54^ Improvement in motor symptoms and QOL has also been observed in adults cancer survivors with neuropathy undergoing endurance and balance training.^55^ Furthermore, there is emerging evidence that exercise therapy, yoga, and acupuncture may improve motor and sensory symptoms.^56^, ^57^, ^58^ Our findings support evaluation of these interventions in survivors of childhood CNS tumors.

The lack of association between motor and sensory impairment and impaired physical function was unexpected. This finding differs from other studies that found associations of motor and sensory impairments with daily function in survivors of non‐CNS tumors.^7^, ^25^ Among 25,583 survivors of childhood cancer from the Childhood Cancer Survivor Study, neuromuscular dysfunction was associated with impaired activities of daily living.^7^ Among 206 survivors of extremity sarcoma from SJLIFE, motor neuropathy was associated with impaired fitness and limited activities of daily living.^25^ We found that poorer visual/motor and motor processing skills were associated with impaired physical function, suggesting central motor processes were larger contributors to physical function than the motor signs and symptoms measured by the mTNS.^59^, ^60^ This is similar to a study of 365 survivors of acute lymphoblastic leukemia from SJLIFE in which visual‐motor processing speed, but not peripheral sensory impairment, was associated with impaired balance.^61^ Our findings suggest that motor and sensory signs and symptoms, as well as central motor processing, impact QOL and physical function in survivors of CNS tumors, and therefore interventions to improve motor and sensory impairment are needed.

The results of our study should be interpreted in the context of potential limitations in addition to those already mentioned. Survivors were treated at a single institution, and results may not be generalizable to all populations of childhood CNS tumor survivors. Furthermore, St. Jude protocols use lower cumulative doses of vinca alkaloid and platinum agents compared to other medulloblastoma protocols, which could have contributed to the lack of association of platinum agents with motor and sensory impairment and may impact generalizability of our findings.^62^, ^63^, ^64^ However, this sample allowed for in‐depth clinical evaluations in a large, diverse population of survivors of CNS tumors,^65^ that are not available with other datasets. We could also only include survivors who were able to complete an in‐person clinical assessment in this study, which may have limited inclusion of the most impaired survivors. Only four survivors who returned for an assessment were unable to complete their evaluation due to functional limitation, so we do not believe this impacted our findings. Furthermore, survivors who returned for assessments had similar markers of performance status including employment status, independent living status, and physical QOL, to survivors who did not return for assessments, suggesting this was a representative sample. Another potential limitation is that this study included survivors at least 5 years from their cancer diagnosis and does not reflect the prevalence of motor and sensory impairment among those who did not survive 5 years from diagnosis. This may have overestimated or underestimated the true prevalence of this outcome. However, these findings still inform care for an important, growing population of long‐term survivors who survive 5 years. Finally, while we tried to define motor and sensory impairment as signs and symptoms indicative of neuropathy by using a validated instrument administered by an in‐person trained exercise physiologist or physical therapist, it is possible both peripheral and central causes contributed to impairment. Regardless of the etiology, we were still able to demonstrate the high prevalence of motor and sensory impairment in survivors of CNS tumors and its association with impactful daily outcomes that will inform care for this group.

## 5 CONCLUSIONS

Approximately one quarter of survivors of childhood CNS tumors had grade ≥2 motor or sensory impairment on exam, which was associated with impaired QOL. Etoposide and vinca alkaloid exposure were associated with motor impairment, while older age and CSI were associated with sensory impairment. Survivors of CNS tumors should be monitored for motor and sensory impairment, and interventions that target motor impairment may be warranted to improve their QOL.

## AUTHOR CONTRIBUTIONS

**Rozalyn L. Rodwin:** Conceptualization (equal); investigation (equal); methodology (equal); writing – original draft (equal); writing – review and editing (equal). **Fang Wang:** Formal analysis (equal); investigation (equal); methodology (equal); writing – original draft (equal); writing – review and editing (equal). **Lu Lu:** Conceptualization (equal); formal analysis (equal); methodology (equal); writing – original draft (equal); writing – review and editing (equal). **Zhenghong Li:** Formal analysis (equal); methodology (equal). **Deo Kumar Srivastava:** Formal analysis (equal); methodology (equal). **Nicholas S. Phillips:** Investigation (equal); writing – original draft (equal); writing – review and editing (equal). **Raja B. Khan:** Investigation (equal); writing – review and editing (equal). **Tara M. Brinkman:** Investigation (equal); writing – review and editing (equal). **Kevin R. Krull:** Investigation (equal); writing – review and editing (equal). **Frederick A. Boop:** Writing – review and editing (equal). **Gregory T. Armstrong:** Investigation (equal); writing – review and editing (equal). **Thomas E. Merchant:** Investigation (equal); writing – review and editing (equal). **Amar Gajjar:** Investigation (equal); writing – review and editing (equal). **Leslie L. Robison:** Investigation (equal); writing – review and editing (equal). **Melissa M. Hudson:** Investigation (equal); writing – review and editing (equal). **Nina S. Kadan‐Lottick:** Conceptualization (equal); investigation (equal); methodology (equal); supervision (equal); writing – original draft (equal); writing – review and editing (equal). **Kirsten K. Ness:** Conceptualization (equal); data curation (equal); formal analysis (equal); investigation (equal); methodology (equal); resources (equal); supervision (equal); writing – original draft (equal); writing – review and editing (equal).

## FUNDING INFORMATION

Support to St. Jude Children's Research Hospital provided by the National Cancer Institute at the National Institutes of Health [U01 CA195547]; Cancer Center Support grant [P30CA021765], and the American Lebanese Syrian Associated Charities. Additional funding was provided by National Cancer Institute through the Yale Cancer Prevention and Control Training Program [T32 CA250803 to RLR]; William O. Seery Mentored Research Award for Cancer Research, Bank of America, N.A., Trustee to RLR; Hyundai Hope on Wheels Young Investigator Award to RLR; COVID‐19 Fund to Retain Clinical Scientists at Yale, sponsored by the Doris Duke Charitable Foundation award # 2021266, and the Yale Center for Clinical Investigation to RLR, and the CTSA Grant Number KL2 TR001862 from the National Center for Advancing Translational Science (NCATS), a component of the National Institutes of Health (NIH) to RLR. The manuscript's contents are solely the responsibility of the authors and do not necessarily represent the official view of NIH.

## CONFLICT OF INTEREST STATEMENT

None declared.

## Supporting information


Tables S1–S4.

## Data Availability

The raw data are available in the St. Jude cloud and can be accessed at https://sjlife.stjude.org/data‐sharing.html and https://www.stjude.cloud/.^66^
