# Supplementary material for: Motor and sensory impairment in survivors of childhood central nervous system (CNS) tumors in the St. Jude Lifetime Cohort (SJLIFE)
Source: Cancer Med. 2024 Jul 26;13(14):e7422. doi: 10.1002/cam4.7422 (PMC11273544; doi:10.1002/cam4.7422)
Supplement: Supplementary file 1 — Tables S1–S4. [file CAM4-13-e7422-s001.docx]

**Supplemental Table 1: Grading of motor and sensory impairment using the modified version of the National Cancer Institute’s Common Terminology Criteria for Adverse Events (CTCAE) version 4.03^1^**

| **Motor Impairment** | |
| --- | --- |
| **Grade** | **Criteria** |
| Grade 0 | - No motor symptoms by scripted interview - Normal strength by manual muscle examination |
| Grade 1 | - Presence of motor symptoms or impaired strength by manual muscle examination of fingers, toes, ankles and wrists - Normal hand and foot strength by dynamometer |
| Grade ≥2 | - Presence of motor symptoms or impaired strength by manual muscle examination   **AND**   - Hand strength by dynamometer (average of both sides) >1.5 standard deviations below community average   **OR**   - Foot strength by dynamometer (average of both sides) >1.5 standard deviations below community average |
| **Sensory Impairment** | |
| **Grade** | **Criteria** |
| Grade 0 | - No sensory symptoms by scripted interview - Normal vibration and pin-prick sensation |
| Grade 1 | - Presence of sensory symptoms, impaired vibration sensation, or impaired pin-prick sensation - Normal balance and writing speed |
| Grade ≥2 | - Presence of self-reported sensory symptoms, impaired vibration sensation, or impaired pin-prick sensation   **AND**   - Abnormal balance in sensory organization test (composite score ≤70) without dysarthria/nystagmus   **OR**   - Slow writing speed (> 10 seconds to write a short sentence) without dysarthria/nystagmus |

**Supplemental Table 2: Prevalence of motor and sensory impairment in survivors and community matched controls by grade of impairment**

|  | **Survivors**  **n (%)** | **95% CI** | **Controls**  **n (%)** | **95% CI** | **P Value^a^** |
| --- | --- | --- | --- | --- | --- |
| Motor Impairment |  |  |  |  |  |
| Grade 0 | 307 (81.2) | 77.3-85.2 | 440 (98.9) | 97.9-99.9 | <.001 |
| Grade 1 | 19 (5.0) | 2.8-7.2 | 1 (0.2) | 0.0-0.7 |  |
| Grade ≥2 | 51 (13.5) | 10.1-16.9 | 4 (0.9) | 0.0-1.8 |  |
| Grade 2 | 29 (7.7) | 5.0-10.4 | 4 (0.9) | 0.0-1.8 |  |
| Grade 3 | 22 (5.8) | 3.5-8.2 | 0 (0.0) | - |  |
| Grade 4 | 0 (0.0) | - | 0 (0.0) | - |  |
| Missing | 1 (0.3) | 0.0-0.8 | 0 (0.0) | - |  |
| Sensory Impairment |  |  |  |  |  |
| Grade 0 | 273 (72.2) | 67.7-76.7 | 387 (87.4) | 83.8-90.1 | <.001 |
| Grade 1 | 39 (10.3) | 7.3-13.4 | 46 (10.4) | 7.5-13.2 |  |
| Grade ≥2 | 64 (16.9) | 13.2-20.7 | 10 (2.3) | 0.9-3.6 |  |
| Grade 2 | 41 (10.9) | 7.7-14.0 | 10 (2.3) | 0.9-3.6 |  |
| Grade 3 | 23 (6.1) | 3.7-8.5 | 0 (0.0) | - |  |
| Grade 4 | 0 (0.0) | - | 0 (0.0) | - |  |
| Missing | 2 (0.5) | 0.0-1.3 | 2 (0.5) | 0.0-1.1 |  |

^a^ P values were calculated using Fisher’s Exact test with missing values excluded. P values remained <.001 when comparing grade 0-1 versus grade ≥2 impairment.

**Supplemental Table 3: Frequency of grade 2 or higher motor and sensory impairment by histologic diagnosis**

| **Histology** | **Motor Impairment** | | **Sensory Impairment** | |
| --- | --- | --- | --- | --- |
|  | **Yes (N=51)** | **No (N=326)** | **Yes (N=64)** | **No (N=312)** |
| Astroglial Tumor (n,%) | 19 (10.3%) | 166 (89.7%) | 19 (10.3%) | 165 (89.7%) |
| Craniopharyngioma (n,%) | 1 (3.5%) | 28 (96.6%) | 1 (3.5%) | 28 (96.6% |
| Ependymoma (n,%) | 9 (22.0%) | 32 (78.1%) | 7 (17.1%) | 34 (82.9%) |
| Germ Cell Tumor (n,%) | 1 (8.3%) | 11 (91.7%) | 2 (16.7%) | 10 (83.3%) |
| Medulloblastoma (n,%) | 20 (19.6%) | 82 (80.4%) | 34 (33.3%) | 68 (66.7%) |
| Other (n,%) | 1 (12.5%) | 7 (87.5%) | 1 (12.5%) | 7 (87.5%) |

**Supplemental Table 4:** Association of treatment modality with grade 2 or higher motor and sensory impairment in multivariable models^a^

|  | **Motor Impairment** | | | **Sensory Impairment** | | |
| --- | --- | --- | --- | --- | --- | --- |
|  | (N=51) | | | (N=64) | | |
|  | n (%) | OR (95% CI) | P Value | n (%) | OR (95% CI) | P Value |
| No treatment or surgery only | 12 (23.5) | 1.00 |  | 9 (14.1) | 1.00 |  |
| Focal radiation^b^ | 12 (23.5) | 0.97 (0.42 - 2.27) | .942 | 14 (21.9) | 1.30 (0.52 - 3.29) | .576 |
| Chemotherapy (with or without focal radiation)^b^ | 11 (21.6) | 1.54 (0.64 - 3.72) | .339 | 12 (18.8) | 2.40 (0.95 - 6.05) | .065 |
| CSI^b^ | 3 (5.9) | 1.47 (0.37 - 5.79) | .583 | 3 (4.7) | 2.16 (0.52 - 8.88) | .288 |
| Chemotherapy, CSI, and surgery | 13 (25.5) | 1.62 (0.69 - 3.77) | .266 | 26 (40.6) | **5.72 (2.49 - 13.14)** | **<.001** |

^a^No variables were selected in the models for motor impairment or sensory impairment. Treatment was forced into the models to estimate associations.

^b^Included survivors who received and did not receive surgery

Abbreviations: CSI, craniospinal radiation

**References:**

1. Hudson MM, Ehrhardt MJ, Bhakta N, et al. Approach for Classification and Severity Grading of Long-term and Late-Onset Health Events among Childhood Cancer Survivors in the St. Jude Lifetime Cohort. *Cancer Epidemiol Biomarkers Prev*. May 2017;26(5):666-674. doi:10.1158/1055-9965.Epi-16-0812
